# Influence of Men's Personality and Social Support on Treatment Decision-Making for Localized Prostate Cancer

**DOI:** 10.1155/2017/1467056

**Published:** 2017-07-12

**Authors:** Elyse Reamer, Felix Yang, Margaret Holmes-Rovner, Joe Liu, Jinping Xu

**Affiliations:** ^1^School of Medicine, Department of Family Medicine and Public Health Sciences, Wayne State University, Detroit, MI, USA; ^2^Department of Medicine, Michigan State University, East Lansing, MI, USA; ^3^School of Medicine, Department of Anesthesiology, Wayne State University, Detroit, MI, USA

## Abstract

**Background:**

Optimal treatment for localized prostate cancer (LPC) is controversial. We assessed the effects of personality, specialists seen, and involvement of spouse, family, or friends on treatment decision/decision-making qualities.

**Methods:**

We surveyed a population-based sample of men ≤ 75 years with newly diagnosed LPC about treatment choice, reasons for the choice, decision-making difficulty, satisfaction, and regret.

**Results:**

Of 160 men (71 black, 89 white), with a mean age of 61 (±7.3) years, 59% chose surgery, 31% chose radiation, and 10% chose active surveillance (AS)/watchful waiting (WW). Adjusting for age, race, comorbidity, tumor risk level, and treatment status, men who consulted friends during decision-making were more likely to choose curative treatment (radiation or surgery) than WW/AS (OR = 11.1, *p* < 0.01; 8.7, *p* < 0.01). Men who saw a radiation oncologist in addition to a urologist were more likely to choose radiation than surgery (OR = 6.0, *p* = 0.04). Men who consulted family or friends (OR = 2.6, *p* < 0.01; 3.7, *p* < 0.01) experienced greater decision-making difficulty. No personality traits (pessimism, optimism, or faith) were associated with treatment choice/decision-making quality measures.

**Conclusions:**

In addition to specialist seen, consulting friends increased men's likelihood of choosing curative treatment. Consulting family or friends increased decision-making difficulty.

## 1. Introduction

Approximately 13% of men in the US will be diagnosed with prostate cancer at some point in their lifetime [1]. Over 80% of prostate cancers are diagnosed at the local stage [2]. The 5-year survival for localized prostate cancer (LPC) is 99% [1]. Three main options are generally available for the treatment of LPC: active surveillance/watchful waiting (AS/WW), surgery (radical prostatectomy), and radiation (internal or external radiation) [3]. Since mortality is essentially the same for each treatment [4], experts recommend that treatment choice should be responsive to patient preferences [5]. These personal preferences have been shown to be shaped by a patient's own beliefs, personality traits [6–8], and the people that he interacts with during the decision-making process [7, 9–20], though many of these studies were performed in majority white populations. Understanding how men's personality traits and social influences impact the treatment decision-making process in a diverse population is important for physicians and other healthcare professionals to provide the best support possible for individual patients as they choose the best treatment for their unique circumstances.

Social influences on decision-making studied previously include consulting friends and family in addition to healthcare providers. Partners and spouses often are involved in discussions about LPC treatment choices with both patients and providers and in choosing the final LPC treatment option [9–11]. Being married or cohabitating was reported to be associated with less decisional conflict and less decision-making difficulty [7]. Being married was also found to be positively associated with choosing curative treatment for LPC, specifically prostatectomy [12], and negatively associated with choosing AS/WW [13]. Family and friends were reported to often urge curative treatment as well [14]. Several studies have found that physician recommendation is the most important factor in a patient's treatment choice [17–19]. However, additional, systematic research examining all the social influences and their impact not only on treatment choice but also on the treatment decision-making process is needed.

Our study sought to further evaluate the important associations between personality traits, social influences, and the LPC treatment decision-making process in a population-based, racially diverse sample. Specifically, we evaluated the effect of personality traits (optimism, pessimism, and faith), physician specialty, and social support consulted (family, friends, and spouse/partner) on patients' LPC treatment choice and qualities of the treatment decision-making process (i.e., decision-making difficulty, satisfaction, and regret).

## 2. Materials and Methods

We conducted a population-based cross-sectional survey of black and white men living in the Metropolitan Detroit area aged 75 years or less and newly diagnosed with LPC between 2009 and 2010. A detailed description of the study method, sampling, and survey instrument has been previously reported [19]. Briefly, new LPC cases were identified by Rapid Case Ascertainment (RCA) in the Metropolitan Detroit Cancer Surveillance System (MDCSS), a population-based cancer registry that is part of the National Cancer Institute's Surveillance, Epidemiology, and End Results (SEER) program. If the patient's physician stated that the patient was healthy enough to participate, the eligible case was mailed a self-administered survey with a small ($10) monetary incentive. The content and design of the surveys were developed based on thorough literature review and refined by the findings of qualitative studies [21, 22]. The Dillman method was used to encourage survey response [23]. To reduce the participant burden, the survey was divided into 2 parts and mailed to participants approximately one month apart. The first part of the survey asked men to report their treatment choice, reasons for the choice, type of specialists seen, and what treatment options were offered and recommended by their physicians [19]. The second half of the survey asked about personality traits (e.g., optimism, pessimism, and faith), who the patient consulted besides physicians, including spouse/partner, other family members, and friends, and decision-making experiences (i.e., decision-making difficulty, satisfaction, and regret) [23, 24]. LPC was defined as T1 to T2 tumors based on American Joint Committee on Cancer (AJCC) stage criteria. The study received approval from the institutional review board at Wayne State University.

### 2.1. Sampling

**Figure 1:**
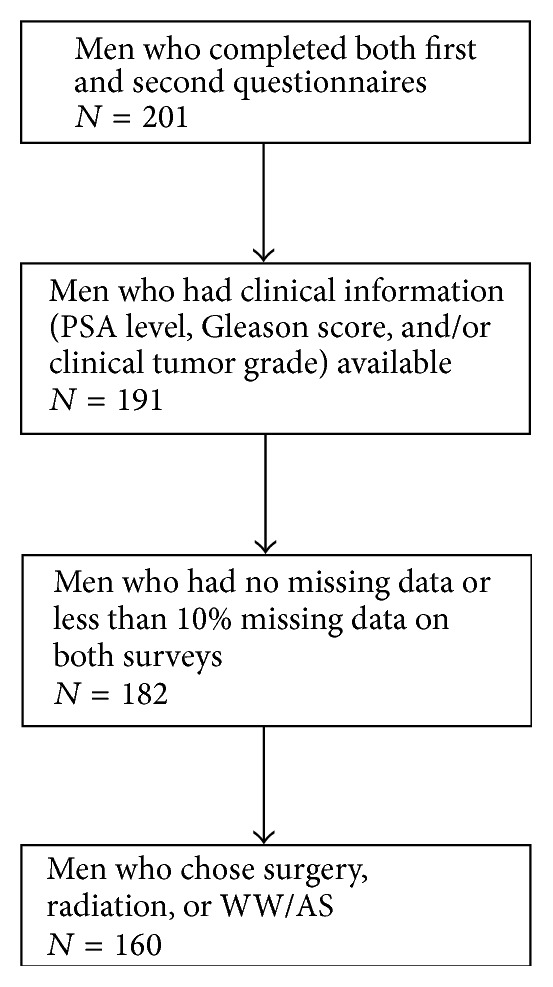
Flowchart of participants included in final analysis.

During the study period, a total of 874 potentially eligible LPC cases were identified. To achieve similar numbers of white and black men, white men were sampled at a ratio of 1 : 3, leaving a total of 559 men sampled for study contact. After initial physician and patient contact, 168 total patients were excluded from the study (118 because their physicians did not approve their participation and 50 because they did not meet all study inclusion criteria), resulting in 391 eligible cases to be surveyed [19]. Of them, 266 men completed the first part of the survey, resulting in a response rate of 68%. 22 men declined the invitation to participate in the second part of the survey. Therefore, a total of 244 men were mailed a second survey, and 201 men completed it with response rate of 82%. Among the 201 men who responded to both surveys, 10 men were excluded from the analysis due to insufficient clinical information (i.e., inability to assess their tumor risk level due to a missing PSA level or Gleason score). Another 9 were excluded due to extensive missing data (missing > 10% of data on both surveys), and another 22 were excluded due to having chosen a treatment option other than surgery, radiation, or WW/AS (Figure 1). A final sample of 160 participants was included in the data analyses for this report. 86% of the 160 participants completed the first survey within six months of diagnosis (mean: 119 days, SD: 54 days); 72% completed the second survey within six months of diagnosis (mean: 158 days, SD: 63 days).

### 2.2. Instruments and Measures

The primary outcome variables were treatment choice and qualities of the treatment decision-making process. Treatment choice was self-reported and included WW/AS, surgery, and radiation. The treatment decision-making qualities (i.e., decision-making difficulty, satisfaction, and regret) were based on existing scales (*α* = 0.87, *α* = 0.86, and *α* = 0.81–0.92, resp.) modified for our study [6, 24–26], with a Cronbach's alpha value of 0.77, 0.75, and 0.74, respectively, in our study. All were measured as 5-point Likert-type responses ranging from “strongly disagree” to “strongly agree.” Higher scores represent more decision-making difficulty, satisfaction, or regret.

Predictor variables included age, self-reported race (black or white), self-reported number of comorbidities, tumor characteristics, education level, presence of a spouse/partner, whether family, friends, or spouse/partner were consulted about the patient's treatment decision, types of physicians seen (urologist, urologist and primary care physician (PCP), and radiation oncologist with or without urologist/PCP), and personality traits (optimism, pessimism, and faith). PSA, Gleason scores, and tumor clinical stage were used to define the tumor risk level according to the American Urological Association-endorsed D'Amico criteria. Self-reported PSA and Gleason scores were used when available and supplemented by MDCSS. The personality trait scale was modified from two preexisting validated scales (*α* = 0.78 and *α* = 0.67–0.86) [27, 28]. Respondents ranked how well each statement matched their personal beliefs using a 5-point Likert-type response format ranging from “not at all true” to “completely true.” Higher scores represented stronger match of each statement to the respondent's beliefs. Factor analysis identified 3 well-defined, meaningful subthemes in the personality trait subscale, optimism, pessimism, and faith score, with a Cronbach alpha of 0.90, 0.69, and 0.76, respectively.

### 2.3. Statistical Analysis

The distribution of demographic characteristics (age, race, marital status, and education), clinical characteristics (PSA level, Gleason score, and number of comorbidities), social influence sources consulted, physicians seen, and personality trait variables was described. Racial differences in the distribution of these variables and their unadjusted effects on treatment choice were examined using chi-square test or Fisher's exact test for dichotomous variables and *t*-tests or ANOVA for continuous variables. Multinomial logistic regression models were performed to examine the effect of each significant predictor in bivariate analysis on treatment choice while adjusting for age, race, tumor risk level, number of comorbidities, and whether treatment had been started or received at the time of survey. Due to the lack of variability in the Likert scale responses for decisional satisfaction and regret, linear regression was not feasible. Instead, the decision-making quality measures were dichotomized with the median as the cutoff. Logistic regression models were then performed to examine the effects of social sources consulted, personality traits, and types of physicians seen on decision-making quality measures (i.e., decision-making difficulty, satisfaction, and regret) while adjusting for age, race, tumor risk level, number of comorbidities, and treatment status. All analyses were computed using R version 3.1.2 (R Development Core Team, Vienna, Austria) with a *p* value < 0.05 being significant.

## 3. Results

Among the 160 men eligible for this study, 59% chose surgery, 31% chose radiation, and 10% chose AS/WW. 103 (64%) of respondents had started or received treatment at the time of survey. Among these men, the mean time between diagnosis and treatment was 57 (±SD 39) days. Significant differences existed between white men and black men in univariate analysis. Compared to white men, black men were more likely to consult their family for treatment decision (66% versus 43%; *p* < 0.01), be unmarried/not partnered (31% versus 10%; *p* < 0.01), have no more than a high school education (76% versus 53%; *p* < 0.01), and report a higher faith score (mean: 3.9 versus 3.1 on a 5-point scale; *p* < 0.01) (Table 1). Overall, men in our sample were highly satisfied (median score: 5.0 on a 5-point scale, SD: 0.4) and had little regret (median score: 1.0 on a 5-point scale, SD: 0.8) with their treatment decision-making process. Men experienced a moderate level of decision-making difficulty (median score: 2.2 on a 5-point scale, SD: 1.0). Using multinomial logistic regression adjusting for age, race, comorbidities, tumor risk level, and treatment status, men who consulted friends regarding their treatment decision were more likely to choose curative treatment (radiation or surgery) compared to WW/AS (radiation [OR = 11.1, *p* < 0.01] or surgery [OR = 8.7, *p* < 0.01]) (Table 2). In addition, when comparing men who saw only a urologist, men who saw a radiation oncologist in addition to a urologist and/or a PCP were more likely to choose radiation compared to surgery (OR = 6.0, *p* = 0.04) (Table 2). Men who consulted family or friends experienced higher decision-making difficulty than men who did not (OR = 2.6, *p* < 0.01, and OR = 3.7, *p* < 0.01, resp.) (Table 3). Consulting one's spouse/partner did not affect decision-making difficulty, satisfaction, or regret (Table 3). Personality traits (optimism, pessimism, and faith) were not associated with treatment choice or with qualities of the treatment decision-making process.

## 4. Discussion

This population-based study evaluated the impact of both social and personality factors on treatment choices and decision-making qualities. We found that social, but not personality, factors predicted treatment choice and decision-making difficulty. These findings underscore the importance of providing decision support not just to patients but also to members of their social support system, including friends, family, and spouse/partner. The previously identified importance of physicians taking patient preferences into account [29] should be expanded to include the opinions and preferences of patient's friends and family members in helping patients make an informed treatment decision for LPC.

An interesting finding of our study was that consultation with friends during decision-making increased men's likelihood of choosing curative treatment compared to WW/AS after adjusting for age, race, comorbidities, tumor risk level, and treatment status. This suggests that friends may encourage patients to choose more aggressive treatment. This broader understanding of the influence of members from the patient's social support networks, while understudied, is consistent with previous findings. Earlier interviews of prostate cancer patients in the UK found that men often felt considerable pressure from family, as well as from doctors and support groups, to pursue curative treatment [14]. A recent focus group study of physicians found that, even with the increase in recommendations of AS/WW as a treatment strategy, most family members and spouses were more often in support of active treatment and opposed to AS [20]. Our recent focus group study found that men and their partners often felt it was necessary to justify their AS decision to their social support, particularly to alleviate the fears of family and friends about their untreated cancer [11]. Of particular importance is the influence of friends or family members who were previously diagnosed with prostate cancer [21, 22]. One study showed LPC patients who consulted other patients to be half as likely to choose AS/WW as those who did not [13]. We have shown that this cohort of patients underestimates their life expectancy without treatment and overestimates their gain in life expectancy with curative treatments [30]. This bias may be shared or influenced by similar misconceptions among family and friends. The recent physician focus group study argued that, even with an increase in patients and physicians willing to choose AS in recent years, patients' family and friends may lack understanding about AS and be more anxious about the untreated cancer than the patient himself [20]. Further educational intervention about LPC treatment choices, particularly about AS, which includes family and friends in addition to patients and their spouses/partners may be needed.

A novel finding of our study was that consulting friends and family was associated with greater difficulty in making a treatment decision. In this study, family did not include patient's spouse/partner. We cannot be certain whether this association occurred because men who are having difficulty making a treatment decision were more likely to turn to their family and friends for advice or because consulting friends and family caused increased decision-making difficulty. Part of the greater decision-making difficulty may be due, in part, to conflicting opinions and preferences among family and friends involved in the decision-making process. The potential for positive social support during this difficult time, however, remains high. A previous study found that discussing treatment options with family or friends, prior to beginning treatment for prostate cancer, significantly improved patients' general happiness at 1 and 6 months following treatment [16]. Some evidence exists to support the use of decision aids among family and friends as a possible solution to ameliorate potential misconceptions held by family and friends. In particular, decision aids with expressed probabilities and explicit values clarifications helped people to have more accurate risk perceptions and to choose a treatment most congruent with their personal beliefs [31]. While these findings come from studies focused on patients, future research should expand the subject population to include patients' family and friends.

Our study did not find a significant association between a man consulting his spouse/partner and treatment choice or qualities of the treatment decision-making process. Previous research has demonstrated that spouses/partners often are involved in discussions about LPC. Frequent roles of spouses/partners are to provide emotional support, discuss treatment options with the patient, go to doctor appointments with the patient and be involved with conversations with the providers, gather information for the patient, aid in sharing information about the diagnosis with family members and friends, and help the patient decide on a treatment choice [9–11]. However, although spouses/partners are often actively involved in the treatment decision-making process, some research argues that they ultimately support or are satisfied with whatever treatment decision the LPC patient makes [10, 11]. Perhaps this may help explain why consulting a spouse is not significantly associated with the final treatment decision. It is also possible that only certain roles that a spouse/partner fills during the LPC treatment decision-making process influence qualities of the treatment decision-making process or final treatment choice.

Consistent with literature, we also found that physician specialty affected treatment choice. Men who saw a radiation oncologist in addition to a urologist and/or a PCP were more likely to choose radiation as compared to surgery after adjusting for age, race, comorbidities, tumor risk level, and treatment status. Such an association is not unexpected, as there are recognized preferences held by each physician specialty. Urologists often recommend surgery [32] or, increasingly recently, AS/WW [33] as the optimal treatment strategy, while radiation oncologists prefer radiation therapy [32]. Jang et al. [34] examined the association between provider visits and treatment choice in 85,088 men with newly diagnosed early-stage prostate cancer. There was a strong association between the type of specialist seen and primary therapy received. A study of 167 LPC patients by Sommers et al. concluded that it is likely that the association between physician specialty and LPC treatment choice reflects both patient preferences and physician bias toward the treatment options offered by their specialty [35]. Two more recent studies confirmed that physician recommendation influenced treatment choice [36, 37]. In addition, it was found that men expressing a preference for AS were more likely to have received a physician recommendation for AS and less likely to have received a recommendation for active therapy [37]. Our finding, which reinforces the association between physician specialty and LPC treatment choice, is important as it stresses the highly influential role that physicians have in patients' treatment decision-making process. Optimal decision-making therefore must openly address physician preferences and biases.

Contradictory to previous literature, we found that faith score was not significantly associated with treatment choice or qualities of the treatment decision-making process. Two reports based on a sample of a LPC patient cohort found that increased spirituality was associated with greater decisional satisfaction, less decisional conflict, less decision-making difficulty, and less decisional regret [7, 8]. In addition, increased spirituality was shown to be associated with increased physical and mental health of men with prostate cancer, including improvement in emotional well-being and decrease in symptom distress and anxiety [38]. These studies measured spirituality using the Functional Assessment of Chronic Illness Therapy-Spirituality Well-Being Scale (FACIT-Sp), which includes two subscales: peace/meaning (capturing a sense of purpose and meaning in life) and faith (spiritual beliefs) [7, 8, 38]. Mollica et al. found that increased scores on both subscales were associated with decisional qualities [7, 8]. In Krupski et al.'s study, the higher peace/meaning subscale was associated with decisional qualities, while the faith subscale was not [38]. Perhaps the faith score that we measured in this study did not fully capture the specific factors of spirituality that impact the treatment decision-making process. It could also be that the use of religious coping as a resource to handle a prostate cancer diagnosis and the stressful decision-making process differs among different groups of men. For example, a study of men with prostate cancer in Georgia demonstrated that black men and those with lower education, lower income, and more comorbidity reported significantly higher levels of religious coping than other groups [39]. Further studies of the impact of faith on men's treatment decision and the treatment decision-making process are needed.

We did not find that men's personality traits of pessimism or optimism had significant associations with either treatment choice or qualities of the treatment decision-making process, which contradicts one study of 125 LPC patients which found that men with lower optimism were at greater risk for treatment decision-making difficulty and lack of decisional satisfaction [6]. This study found that self-efficacy partially mediated the effect of optimism on treatment decisional quality [6]. Although we used a similar scale to measure optimism, we did not assess self-efficacy in our study. The time point at which the participants in our study were surveyed differed from the previous study, when men were surveyed after choosing but before receiving treatment [6], while we surveyed men who had been diagnosed about 6 months previously regardless of whether they had started their treatment. Furthermore, we had a higher percentage of black men compared to the previous study, which could also contribute to the difference in findings. The complex relationship of personal beliefs, personality traits, and religion/faith and their influence on decision-making needs further investigation.

This is one of few population-based studies that examined the effects of physician specialty, patient personality traits, and social influences on LPC treatment choice and decisional quality outcomes. Despite the novel nature of this study's topic of investigation, there are several limitations to this study. First, as with any survey study, there is potential for recall bias. However, we assessed the degree of accuracy among patient self-reported data by comparing patient-reported tumor characteristics with tumor characteristics from our tumor registry (MDCSS). These two sets of data were highly correlated (*ρ* > 0.7, data not shown) [19]. Any bias resulting from misclassification of some variables due to self-reporting would not likely have differed significantly between the different demographic groups within our study. Second, we oversampled black men to achieve a more racially diverse study sample. It is possible that our study design may have contributed to potential selection bias. Third, our sample was gathered from the Metropolitan Detroit area, so the findings of our study may not be applicable to areas with different populations. However, our study sample was more racially diverse than many other studies examining the treatment decision-making process of LPC patients. Fourth, our study's relatively small sample size limited our ability to perform race-stratified data analysis. Larger, racially diverse studies are needed to confirm our study findings. Our study also had a lower number of men (*n* = 16/160) who chose AS/WW. Larger studies are needed to confirm our findings for men who chose AS/WW, particularly since the number of men being recommended and choosing AS/WW as a treatment for LPC has increased in recent years [34]. We also did not differentiate watchful waiting from active surveillance in this study due to the small sample size and because these terms are often used interchangeably by physicians and patients. As this survey was done during the period from 2009 to 2010, it likely underrepresents AS in present practice. However, the main treatment options and their possible benefits and harms as well as the controversies surrounding the best treatment for individuals are not changed. Finally, our data were skewed toward high satisfaction and low regret with treatment decision with little variability. This may limit our ability in delineating any associations between personality traits or social influences consulted and decisional satisfaction or regret. This may also be due to the short time interval between making a treatment choice and completing our survey. Longer-term studies with larger populations are needed to further explore these associations.

## 5. Conclusion

This population-based study of a racially diverse cohort highlights the important effect of social influences during the patient's treatment decision-making process, including patient's personality traits, family, friends, and physicians, on his treatment choice and decisional quality outcomes. Consulting with friends increased men's odds of choosing curative treatment, and consulting with family and/or friends was associated with an increase in men's difficulty in making a treatment decision. Men who saw a radiation oncologist were more likely to choose radiation than surgery. These findings demonstrate the importance of an informed treatment decision-making process that should include both the patient and their family and friends to align preferences, provide education, and reduce decision-making difficulty. These findings also suggest expanded use of decision aids and other educational interventions to recognize and include family and friends in the shared decision-making process. Developing realistic expectations of treatments across communities of influence may help guide patients to make the treatment choice that best fits their own goals and preferences.

## Figures and Tables

**Table 1:** Differences in demographic, clinical, and personality characteristics by race and treatment choice.

Variable	Total	By race			By treatment choice			
	*n* = 160 (%)	White *n* = 89 (%)	Black *n* = 71 (%)	*p*-value^*∗*^	WW/AS *n* = 16 (%)	Radiation *n* = 50 (%)	Surgery *n* = 94 (%)	*p*-value^*∗*^
Age								
Mean (SD)	61.0 (7.3)	61.8 (6.5)	60.1 (8.2)	0.14	64.6 (7.4)	63.0 (6.9)	59.4 (7.1)	**<0.01**
Less than 65	102 (63.8)	55 (61.8)	47 (66.2)	0.68	8 (50.0)	28 (56.0)	66 (70.2)	0.12
65 and greater	58 (36.3)	34 (38.2)	24 (33.8)		8 (50.0)	22 (44.0)	28 (29.8)	
# of comorbidities								
0	34 (21.3)	22 (24.7)	12 (16.9)	0.42	3 (18.8)	4 (8.0)	27 (28.7)	**0.02**
1	60 (37.5)	31 (34.8)	29 (40.8)		4 (25.0)	21 (42.0)	35 (37.2)	
2	38 (23.8)	23 (25.8)	15 (21.1)		6 (37.5)	11 (22.0)	21 (22.3)	
≥3	28 (17.5)	13 (14.6)	15 (21.1)		3 (18.8)	14 (28.0)	11 (11.7)	
PSA level								
≤4	66 (42.0)	39 (44.3)	27 (39.1)	0.59	4 (26.7)	23 (46.0)	39 (42.4)	0.61
5–9	70 (44.6)	40 (45.5)	30 (43.5)		7 (46.7)	21 (42.0)	42 (45.7)	
10–19	8 (5.1)	3 (3.4)	5 (7.2)		2 (13.3)	2 (4.0)	4 (4.3)	
≥20	13 (8.3)	6 (6.8)	7 (10.1)		2 (13.3)	4 (8.0)	7 (7.6)	
Gleason score								
≤6	80 (50.0)	50 (56.2)	30 (42.3)	0.16	8 (50.0)	30 (60.0)	42 (44.7)	**0.04**
7	65 (40.6)	33 (37.1)	32 (45.1)		4 (25.0)	15 (30.0)	46 (48.9)	
8–10	15 (9.4)	6 (6.7)	9 (12.7)		4 (25.0)	5 (10.0)	6 (6.4)	
Tumor risk Level^†^								
Low	28 (18.3)	17 (19.5)	11 (16.7)	0.79	5 (33.3)	19 (42.2)	4 (4.3)	**<0.01**
Intermediate	44 (28.8)	26 (29.9)	18 (27.3)		8 (53.3)	15 (33.3)	21 (22.6)	
High	81 (52.9)	44 (50.6)	37 (56.1)		2 (13.3)	11 (24.4)	68 (73.1)	
Treatment started/received by survey								
Yes	103 (64.4)	63 (70.8)	40 (56.3)	0.08	5 (31.3)	35 (70.0)	63 (67.0)	**0.02**
No	57 (35.6)	26 (29.2)	31 (43.7)		11 (68.8)	15 (30.0)	31 (33.0)	
Education								
≤High school	101 (63.5)	47 (53.4)	54 (76.1)	**<0.01**	11 (68.8)	35 (70.0)	55 (59.1)	0.40
>High school	58 (36.5)	41 (46.6)	17 (23.9)		5 (31.3)	15 (30.0)	38 (40.9)	
Married/partnered								
Yes	127 (80.4)	79 (89.8)	48 (68.6)	**<0.01**	12 (75.0)	40 (81.6)	75 (80.6)	0.84
No	31 (19.6)	9 (10.2)	22 (31.4)		4 (25.0)	9 (18.4)	18 (19.4)	
Consulted family								
Yes	85 (53.5)	38 (43.2)	47 (66.2)	**<0.01**	6 (37.5)	28 (56.0)	51 (54.8)	0.42
No	74 (46.5)	50 (56.8)	24 (33.8)		10 (62.5)	22 (44.0)	42 (45.2)	
Consulted friends								
Yes	85 (53.5)	48 (54.5)	37 (52.1)	0.88	3 (18.8)	31 (62.0)	42 (45.2)	**<0.01**
No	74 (46.5)	40 (45.5)	34 (47.9)		13 (81.3)	19 (38.0)	51 (54.8)	
Consulted spouse/partner								
Yes	120 (76.9)	65 (74.7)	55 (79.7)	0.59	8 (57.1)	38 (77.6)	74 (79.6)	0.20
No	36 (23.1)	22 (25.3)	14 (20.3)		6 (42.9)	11 (22.4)	19 (20.4)	
Physician seen								
Urologist only	15 (9.2)	11 (13.3)	4 (6.8)	0.47	3 (18.8)	2 (4.3)	10 (12.3)	**<0.01**
Urologist/PCP only	59 (41.5)	33 (37.3)	26 (44.1)		7 (43.8)	5 (10.6)	47 (58.0)	
Rad. onc. ± urologist/PCP	70 (49.3)	41 (49.4)	29 (49.2)		6 (37.5)	40 (85.1)	24 (29.6)	
Optimism^‡^								
Mean (SD)	4.0 (0.8)	3.9 (0.7)	4.0 (0.9)	0.44	4.1 (0.6)	4.2 (0.5)	3.9 (0.9)	0.09
Pessimism^‡^								
Mean (SD)	2.0 (0.8)	1.9 (0.7)	2.0 (0.9)	0.42	1.8 (0.7)	1.9 (0.7)	2.0 (0.8)	0.24
Faith score^‡^								
Mean (SD)	3.5 (1.1)	3.1 (1.1)	3.9 (1.1)	**<0.01**	3.3 (1.2)	3.6 (1.1)	3.4 (1.2)	0.97

^*∗*^ *p* values were calculated using chi-square test or Fisher's exact test for dichotomous data and *t*-tests or ANOVA for continuous outcomes. ^†^Tumor risk level categorized using the American Urological Association endorsed D'Amico criteria: low indicates PSA level < 10, Gleason score ≤ 6, and clinical stage T1-2a; intermediate indicates PSA of 10–20, Gleason score of 7, and clinical stage T2b; high indicates PSA > 20, Gleason score ≥ 8, and clinical stage T2c-3a. ^‡^Measured on a scale of 1 to 5: 1, not at all true, and 5, completely true.

**Table 2:** Factors associated with treatment choice.

Variable	Radiation versus WW/AS		Surgery versus WW/AS		Surgery versus radiation	
	OR (95% CI)^*∗*^	*p*-value^†^	OR (95% CI)^*∗*^	*p*-value^†^	OR (95% CI)^*∗*^	*p*-value^†^
Consulted friends	11.07 (2.21 to 55.3)	**<0.01**	8.67 (1.73 to 43.6)	**<0.01**	0.78 (0.31 to 1.99)	0.61
Physician seen						
Urologist only (ref.)						
Urologist/PCP	0.29 (0.02 to 3.79)	0.35	1.20 (0.18 to 8.17)	0.86	4.12 (0.45 to 3.78)	0.21
Rad. onc. ± urologist/PCP	6.06 (0.74 to 49.4)	0.09	1.01 (0.15 to 6.65)	0.99	0.17 (0.03 to 0.94)	**0.04**

^*∗*^Adjusted for age, comorbidities, tumor risk level, race, and treatment status. ^†^Calculated using multinomial logistic regression.

**Table 3:** Factors associated with treatment decisional quality outcomes.

Variable	Decisional satisfaction		Decision-making difficulty		Decisional regret	
	OR (95% CI)^*∗*^	*p*-value^†^	OR (95% CI)^*∗*^	*p*-value^†^	OR (95% CI)^*∗*^	*p*-value^†^
Consulted family	0.65 (0.32 to 1.29)	0.22	2.59 (1.29 to 5.35)	**<0.01**	1.60 (0.81 to 3.19)	0.18
Consulted friends	0.98 (0.50 to 1.91)	0.94	3.70 (1.86 to 7.66)	**<0.01**	1.79 (0.92 to 3.54)	0.09
Consulted spouse/partner	0.69 (0.29 to 1.57)	0.39	2.23 (0.96 to 5.48)	0.07	1.05 (0.47 to 2.38)	0.91
Optimism	1.36 (0.87 to 2.27)	0.19	0.94 (0.59 to 1.47)	0.77	0.82 (0.51 to 1.28)	0.40
Pessimism	0.79 (0.50 to 1.25)	0.31	1.19 (0.75 to 1.89)	0.46	1.25 (0.79 to 1.98)	0.34
Faith score	1.34 (0.98 to 1.88)	0.08	1.56 (0.85 to 1.58)	0.36	0.97 (0.71 to 1.32)	0.84
Physician seen						
Urologist only (ref.)						
Urologist/PCP	1.74 (0.53 to 5.89)	0.36	0.79 (0.24 to 2.67)	0.69	0.49 (0.14 to 1.62)	0.25
Rad. onc. ± urologist/PCP	1.99 (0.62 to 6.52)	0.24	1.21 (0.38 to 3.98)	0.75	0.39 (0.11 to 1.25)	0.12

^*∗*^Adjusted for age, comorbidities, tumor risk level, race, and treatment status. ^†^Calculated using logistic regression. ^‡^Rad. onc.: radiation oncologist.
